# Nonuniform Exponential Dichotomies in Terms of Lyapunov Functions for Noninvertible Linear Discrete-Time Systems

**DOI:** 10.1155/2013/901026

**Published:** 2013-11-06

**Authors:** Ioan-Lucian Popa, Mihail Megan, Traian Ceauşu

**Affiliations:** ^1^Department of Mathematics, University “1st December 1918” of Alba Iulia, 510009 Alba Iulia, Romania; ^2^Academy of Romanian Scientists, Independenţei 54, 050094 Bucharest, Romania; ^3^Department of Mathematics, Faculty of Mathematics and Computer Science, West University of Timişoara, Vasile Pârvan Boulevard No. 4, 300223 Timişoara, Romania

## Abstract

The aim of this paper is to give characterizations in terms of Lyapunov functions for nonuniform exponential dichotomies of nonautonomous and noninvertible discrete-time systems.

## 1. Introduction

The notion of (uniform) exponential dichotomy essentially introduced by Perron in [1] for differential equations and by Li in [2] for difference equations plays a central role in a substantial part of the asymptotic behaviors theory of dynamical systems.

In some situations, particularly in the nonautonomous settings, the concept of uniform exponential dichotomy is too restrictive and it is important to consider more general behaviors.

One of the main reasons for weakening the assumption of uniform exponential dichotomy is that from the point of view of ergodic theory almost all variational equations in a finite-dimensional space admit a nonuniform exponential dichotomy. On the other hand it is important to treat the case of noninvertible systems because of their interest in applications (e.g., random dynamical systems, generated by random parabolic equations, are not invertible).

The importance of Lyapunov functions is well established in the study of linear and nonlinear systems in both continuous and discrete-time. Thus, after the seminal work of Lyapunov republished most recently in [3] relevant results using Lyapunov's direct method (or second method) are presented in the books due to LaSalle, Lefschetz [4], Hahn [5], Halanay, Wexler [6], and Maliso and Mazenc [7].

This paper considers the general notion of nonuniform exponential dichotomy for nonautonomous linear discrete-time systems in Banach spaces. The main objective is to give characterizations of nonuniform exponential dichotomy in terms of Lyapunov functions for the general case of noninvertible linear discrete-time systems, as a particular case is the concept of (nonuniform) exponential dichotomy for the discrete-time linear systems which are invertible in the unstable directions. This approach can be found in the works of Barreira and Valls (see [8, 9]), and for the uniform approach we can mention the paper of Papaschinopoulos (see [10]).

In the nonuniform exponential dichotomies of linear discrete-time systems presented in this paper we consider two types of projection sequences: invariant and strongly invariant, which are distinct even in the finite-dimensional case.

We remark that we consider linear discrete-time systems having the right hand side not necessarily invertible and the dichotomy concepts studied in this paper use the evolution operators in forward time. In the definition of nonuniform exponential dichotomy we assume the existence of invariant projections sequence. At a first view the existence of such sequence is a strong hypothesis. This impediment can be eliminated using the notion of admissibility (see [11]).

The main theme of our paper is the relation between the notion of nonuniform exponential dichotomy with invariant projection sequences and the notion of Lyapunov functions. The case of exponential dichotomy with strongly invariant projection sequences was considered by Barreira and Valls (see [8, 9]).

## 2. Definitions, Notations, and Preliminary Results

We first fix the notions and introduce the basic concepts underlying this paper. By *ℕ* we denote the positive integers and ℝ_+_ denotes the set of positive real numbers. *X* is a real or complex Banach space and *ℬ*(*X*) is the Banach algebra of all bounded linear operators on *X*. The norm on *X* and *ℬ*(*X*) will be denoted by ||·||. We denote by *I* the identity operator on *X*.

If *A* ∈ *ℬ*(*X*) then we will denote by Ker⁡*A* and by *Im*⁡*A*, respectively, the kernel and range of *A*; that is,

(1) $$\begin{array}{c} \mathrm{Ker}A=\left\{x\in X:Ax=0\right\}, \end{array}$$

respectively,

(2) Im⁡A={Ax:x∈X}.

We also denote by Δ the set of all pairs of natural numbers (*m*, *n*) with *m* ≥ *n*.

Throughout this paper, we consider the linear discrete-time systems of the form

$$\begin{array}{cc} \left(𝔄\right) & x_{n+1}=A_{n}x_{n},\;n\in \mathbb{N}, \end{array}$$

where (*A*_*n*_) is a sequence in *ℬ*(*X*). If for every *n* ∈ *ℕ* the operator (*A*_*n*_) is invertible, then the linear discrete-time system ((*𝔄*)) is called * reversible.* Every solution *x* = (*x*_*n*_) of the system ((*𝔄*)) is given by

(3) $$\begin{array}{c} x_{m}=𝒜\left(m,n\right)x_{n}, \end{array}$$

for all (*m*, *n*) ∈ Δ, where *𝒜* : Δ → *ℬ*(*X*) is defined by

(4) $$\begin{array}{c} 𝒜\left(m,n\right)=\{\begin{array}{cc} A_{m-1}\cdots A_{n} & \text{if}\;\;m>n\\ I & \text{if}\;\;m=n. \end{array} \end{array}$$

The map *𝒜* is called the discrete * evolution operator* associated to the system ((*𝔄*)).

**Remark 1**

The discrete evolution operator *𝒜*(*m*, *n*) satisfies the propagator property; that is,

(5) $$\begin{array}{c} 𝒜\left(m,n\right)𝒜\left(k,n\right)=𝒜\left(m,n\right), \end{array}$$

for all (*m*, *k*), (*k*, *n*) ∈ Δ.

**Definition 2**

A sequence (*P*_*n*_) in *ℬ*(*X*) is called a projections sequence on *X*, if

(6) $$\begin{array}{c} P_{n}^{2}=P_{n},\;\text{for}\;\text{every}\;n\in \mathbb{N}. \end{array}$$

**Remark 3**

If (*P*_*n*_) is a projections sequence on *X* the sequence (*Q*_*n*_) defined by

(7) $$\begin{array}{c} Q_{n}=I-P_{n},\;\forall n\in \mathbb{N} \end{array}$$

is a projections sequence on *X*, which is called the * complementary projections* of (*P*_*n*_). We remark that *P*_*n*_ *Q*_*n*_ = *Q*_*n*_ *P*_*n*_ = 0, Ker⁡*P*_*n*_ = *Im*⁡*Q*_*n*_, and Ker⁡*Q*_*n*_ = *Im*⁡*P*_*n*_.

**Definition 4**

A projection sequence (*P*_*n*_) is called invariant for the system ((*𝔄*)) if

(8) $$\begin{array}{c} A_{n}P_{n}=P_{n+1}A_{n}, \end{array}$$

for all *n* ∈ *ℕ*.

**Remark 5**

In the particular case when ((*𝔄*)) is autonomous, that is, *A*_*n*_ = *A* ∈ *ℬ*(*X*) and *P*_*n*_ = *P* ∈ *ℬ*(*X*) for all *n* ∈ *ℕ*, then (*P*_*n*_) is invariant for ((*𝔄*)) if and only if *AP* = *PA*.

**Remark 6**

The relation (8) from Definition 4 is also true for the complementary projection (*Q*_*n*_) and, as a consequence of (8), we have that

(9) $$\begin{array}{c} 𝒜\left(m,n\right)P_{n}=P_{m}𝒜\left(m,n\right), \end{array}$$

respectively,

(10) $$\begin{array}{c} 𝒜\left(m,n\right)Q_{n}=Q_{m}𝒜\left(m,n\right), \end{array}$$

for all (*m*, *n*) ∈ Δ.

**Remark 7**

If (*P*_*n*_) is a projections sequence invariant for the reversible system ((*𝔄*)) then *𝒜*(*m*, *n*) is invertible for all (*m*, *n*) ∈ *ℕ* ^2^ and

(11) $$\begin{array}{c} 𝒜{\left(m,n\right)}^{-1}P_{m}=P_{n}𝒜{\left(m,n\right)}^{-1},\\ 𝒜{\left(m,n\right)}^{-1}Q_{m}=Q_{n}𝒜{\left(m,n\right)}^{-1}, \end{array}$$

for all (*m*, *n*) ∈ Δ.

**Definition 8**

Let (*P*_*n*_) be a projections sequence which is invariant for the system ((*𝔄*)). We say that (*P*_*n*_) is *strongly invariant* for ((*𝔄*)) if for every (*m*, *n*) ∈ Δ the linear operator *𝒜*(*m*, *n*) is an isomorphism from Ker⁡*P*_*n*_ to Ker⁡*P*_*m*_.

A characterization of strongly invariant projections sequence is given by the following.

**Proposition 9**

Let (*P*_*n*_) be a projections sequence which is invariant for the system ((*𝔄*)). Suppose that for all (*m*, *n*) ∈ Δ the evolution operator *𝒜*(*m*, *n*) is injective on Ker⁡*P*_*n*_. Then (*P*_*n*_) is strongly invariant for ((*𝔄*)) if and only if

(12) Ker⁡Pm⊂Im⁡𝒜(m,n)

for all (*m*, *n*) ∈ Δ.

**Proof**

*Necessity.* If (*P*_*n*_) is strongly invariant for ((*𝔄*)) and *y* ∈ Ker⁡*P*_*m*_ then there is *x* ∈ Ker⁡*P*_*n*_ with *y* = *𝒜*(*m*, *n*)*x* and hence *y* ∈ *Im*⁡*𝒜*(*m*, *n*).

*Sufficiency.* We will prove that for every *y* ∈ Ker⁡*P*_*m*_ there exists *x* ∈ Ker⁡*P*_*n*_ with *y* = *𝒜*(*m*, *n*)*x*.

Let *y* ∈ Ker⁡*P*_*m*_. Then *y* ∈ *Im*⁡*Q*_*m*_ and hence *y* = *Q*_*m*_ *y*. Moreover, from the hypothesis there is *x*_0_ ∈ *X* such that *y* = *𝒜*(*m*, *n*)*x*_0_. Then

(13) $$\begin{array}{c} y=Q_{m}y=Q_{m}𝒜\left(m,n\right)x_{0}=𝒜\left(m,n\right)Q_{n}x_{0}=𝒜\left(m,n\right)x, \end{array}$$

where *x* = *Q*_*n*_ *x*_0_ ∈ Ker⁡*P*_*n*_.

**Corollary 10**

If the projections sequence (*P*_*n*_) is invariant for the reversible system ((*𝔄*)) then it is also strongly invariant for ((*𝔄*)).

An example of invariant projections sequence which is not strongly invariant is given by the following.

**Example 11**

Let *X* = ℝ^3^ with the norm

(14) $$\begin{array}{c} ||\left(x_{1},x_{2},x_{3}\right)||=\left|x_{1}\right|+\left|x_{2}\right|+\left|x_{3}\right| \end{array}$$

and let ((*𝔄*)) be the discrete-time system defined by the sequence

(15) $$\begin{array}{c} A_{n}\left(x_{1},x_{2},x_{3}\right)=\{\begin{array}{cc} \left(\frac{x_{1}}{e},ex_{2},ex_{3}\right) & \text{if}\;n\geq 1\\ \left(x_{1},0,x_{3}\right) & \text{if}\;n=0. \end{array} \end{array}$$

It is easy to verify that the sequence (*P*_*n*_) defined by

(16) $$\begin{array}{c} P_{n}\left(x_{1},x_{2},x_{3}\right)=\{\begin{array}{cc} \left(x_{1},x_{2},0\right) & \;\text{if}\;n=0\\ \left(x_{1}+x_{2}e^{-2n},0,0\right) & \;\text{if}\;n\geq 1 \end{array} \end{array}$$

is a projections sequence which is invariant for the system ((*𝔄*)). Moreover, the evolution operator associated to the system ((*𝔄*)) is given by

(17) 𝒜(m,n)(x1,x2,x3)  ={(x1en−m,x2em−n,x3em−n)if  m≥n≥1or  m=n=0(x1e−m,0,x3em)  if  m>n=0

for all (*m*, *n*) ∈ Δ. We can see that the evolution operator is injective on Ker⁡*P*_*n*_. The sequence (*P*_*n*_) is not strongly invariant for ((*𝔄*)) because *y* = (−1/*e* ^2^, 1,0) ∈ Ker⁡*P*_1_ and *y* ∉ *Im*⁡*𝒜*(1,0).

**Remark 12**

If the projections sequence (*P*_*n*_) is strongly invariant for the system ((*𝔄*)) then there exist *ℬ* : Δ → *ℬ*(*X*) such that for all (*m*, *n*) ∈ Δ the evolution operator *ℬ*(*m*, *n*) is an isomorphism from Ker⁡*P*_*m*_ to Ker⁡*P*_*n*_.

**Proposition 13**

The function *ℬ* : Δ → *ℬ*(*X*) has the following properties:

*(b*_*1*_) *𝒜*(*m*, *n*)*ℬ*(*m*, *n*)*Q*_*m*_ = *Q*_*m*_,
*(b*_*2*_) *ℬ*(*m*, *n*)*𝒜*(*m*, *n*)*Q*_*n*_ = *Q*_*n*_,
*(b*_*3*_) *ℬ*(*m*, *n*)*Q*_*m*_ = *Q*_*n*_ *ℬ*(*m*, *n*)*Q*_*m*_,
*(b*_*4*_) *Q*_*m*_ = *ℬ*(*m*, *m*)*Q*_*m*_ = *Q*_*m*_ *ℬ*(*m*, *m*)*Q*_*m*_

for all (*m*, *n*) ∈ Δ.

**Proof**

The properties (b_1_) and (b_2_) are immediate.

(b_3_) We observe that for every (*m*, *n*, *x*) ∈ Δ × *X* we have that

(18) Qmx∈Im⁡Qm=Ker⁡Pm

and hence

(19) ℬ(m,n)Qmx∈Ker⁡Pn=Im⁡Qm,

which implies (b_3_).

(b_4_) follows immediately from (b_1_) and (b_3_).

**Remark 14**

If the projections sequence (*P*_*n*_) is invariant for the reversible system ((*𝔄*)) then

(20) $$\begin{array}{c} ℬ\left(m,n\right)=𝒜{\left(m,n\right)}^{-1}, \end{array}$$

for all (*m*, *n*) ∈ Δ.

## 3. Nonuniform Exponential Dichotomies

 In this section we investigate some dichotomy concepts of linear discrete-time systems ((*𝔄*)) with respect to a projections sequence (*P*_*n*_) invariant for ((*𝔄*)).

**Definition 15**

We say that system ((*𝔄*)) admits a *nonuniform exponential dichotomy* (n.e.d.) with respect to the projections sequence (*P*_*n*_), if there exist a constant *α* > 0 and a function *D* : *ℕ* → [1, *∞*) such that the following properties hold:

(21) $$\begin{array}{c} e^{\alpha (m-n)}||𝒜\left(m,n\right)x||\leq D\left(n\right)||x||, \end{array}$$

(22) $$\begin{array}{c} e^{\alpha (m-n)}||y||\leq D\left(m\right)||𝒜\left(m,n\right)y||, \end{array}$$

for all (*m*, *n*) ∈ Δ and all (*x*, *y*) ∈ *Im*⁡*P*_*n*_ × Ker⁡*P*_*n*_.

As particular cases of nonuniform exponential dichotomy, we have the following.

1. If *D*(*n*) = *ce* ^*εn*^ with *c* ≥ 1 and *ε* ≥ 0 then we say that system ((*𝔄*)) admits an *exponential dichotomy* (e.d.).
2. If *D*(*n*) = *D* ≥ 1 for all *n* ∈ *ℕ* then we say that system ((*𝔄*)) is * uniformly exponentially dichotomic* (u.e.d.).

**Remark 16**

If *D*(*n*) = *D* ≥ 1 for all *n* ∈ *ℕ* and (*P*_*n*_) is bounded (i.e., there exist *M* ≥ 1 such that ||*P*_*n*_|| ≤ *M*) then it can be easily checked that the concept of nonuniform exponential dichotomy considered in our paper is in fact a particular case of exponential forward splitting considered in [12], with (*m*, *n*) ∈ Δ.

**Remark 17**

If the system ((*𝔄*)) is uniformly exponentially dichotomic and (*P*_*n*_) is bounded then the sequence *A*_*n*_ is uniformly bounded with respect to *n*. (i.e., sup⁡_*n*∈*ℕ*_⁡||*A*_*n*_|| < *∞*). On the other hand, sup⁡_*n*∈*ℕ*_⁡||*A*_*n*_|| may be finite and system ((*𝔄*)) admits a nonuniform behavior. See [13] for various examples for nonuniform exponential contractions and [14, 15] for nonuniform dichotomies. For the differential equations case, see [16].

**Remark 18**

The connection between the dichotomy concepts considered in this paper can be synthesized as (u.e.d.) *⇒* (e.d.) *⇒* (n.e.d.). The following examples show that the converse implications are not valid.

**Example 19**

Let ((*𝔄*)) be the linear discrete-time system and (*P*_*n*_) the projections sequence considered in Example 11. By a simple computation we can see that

(23) $$\begin{array}{c} e^{m-n}||𝒜\left(m,n\right)x||\leq e^{2n}||x||, \end{array}$$

respectively,

(24) $$\begin{array}{c} e^{m-n}||y||\leq e^{2m}||𝒜\left(m,n\right)y|| \end{array}$$

for all (*m*, *n*) ∈ Δ and all (*x*, *y*) ∈ *Im*⁡*P*_*n*_ × Ker⁡*P*_*n*_. Hence, for *α* = 1 and *D*(*n*) = *e* ^2*n*^ the system ((*𝔄*)) is nonuniform exponentially dichotomic. Obviously, the nonuniform part cannot be removed.

**Example 20**

Let *X* = *l* ^*∞*^(ℝ) be the Banach space of bounded real-valued sequences, endowed with the norm

(25) $$\begin{array}{c} ||x||=\underset{n\geq 0}{\sup}\left|x_{n}\right|,\;\text{for}\;x=\left(x_{0},x_{1},\ldots ,x_{n},\ldots \right)\in X. \end{array}$$

Let (*P*_*n*_) be a sequence in *ℬ*(*X*) defined by

(26) Pn(x0,x1,x2,…)  =(x0+(2n2−1)x1,0,x2+(2n2−1)x3,0,…).

It is a simple verification to see that (*P*_*n*_) is a projections sequence with the complementary

(27) $$\begin{array}{c} Q_{n}\left(x_{0},x_{1},x_{2},\ldots \right)=\left(\left(1-2^{n^{2}}\right)x_{1},x_{1},\left(1-2^{n^{2}}\right)x_{3},x_{3},\ldots \right). \end{array}$$

We consider the linear discrete-time system ((*𝔄*)) defined by the sequence (*A*_*n*_) given by

(28) $$\begin{array}{c} A_{n}=2^{a_{n}-a_{n+1}}P_{n}+2^{a_{n+1}-a_{n}}Q_{n+1}, \end{array}$$

where

(29) $$\begin{array}{c} a_{n}=\{\begin{array}{cc} \frac{2k}{3} & \text{if}\;n=2k\\ 2k+1 & \text{if}\;n=2k+1, \end{array} \end{array}$$

for all *n* ∈ *ℕ* and all *x* ∈ *X*.

We have that the evolution operator associated to ((*𝔄*)) is

(30) $$\begin{array}{c} 𝒜\left(m,n\right)=2^{a_{n}-a_{m}}P_{n}+2^{a_{m}-a_{n}}Q_{n}, \end{array}$$

for all (*m*, *n*) ∈ Δ.

We observe that for all (*m*, *n*) ∈ Δ we obtain

(31) $$\begin{array}{c} a_{n}-a_{m}\leq \frac{n-m}{3}+\frac{2n}{3} \end{array}$$

hence

(32) $$\begin{array}{c} a_{m}-a_{n}\geq \frac{m-n}{3}-\frac{2n}{3}. \end{array}$$

We can see that (*P*_*n*_) is strongly invariant for the system ((*𝔄*)) and

(33) $$\begin{array}{c} ||𝒜\left(m,n\right)x||\leq 2^{2n/3}2^{-(1/3)(m-n)}||x|| \end{array}$$

and, respectively,

(34) $$\begin{array}{c} ||ℬ\left(m,n\right)y||\leq 2^{(2/3)m}2^{-(1/3)(m-n)}||y|| \end{array}$$

for all (*m*, *n*) ∈ Δ and all (*x*, *y*) ∈ *Im*⁡*P*_*n*_ × Ker⁡*P*_*n*_. Finally, we observe that system ((*𝔄*)) is exponentially dichotomic.

On the other side, if we suppose that the system ((*𝔄*)) admits a uniform approach, taking into account (21) from Definition 15 with *D*(*n*) = *D* and (33) for *m* = *n* + 1 and *n* = 2*k* + 1, we have that

(35) $$\begin{array}{c} 2^{\left(4k+1\right)/3}||x||\leq D||x|| \end{array}$$

for all *x* ∈ *Im*⁡*P*_*n*_, which shows that nonuniform part cannot be removed.

**Example 21**

Consider, on *X* = ℝ^2^, the sequence (*A*_*n*_) in *ℬ*(ℝ^2^) given by

(36) $$\begin{array}{c} A_{n}\left(x_{1},x_{2}\right)=\left(ea_{n}x_{1},\frac{x_{2}}{e}\right) \end{array}$$

for all (*n*, *x*_1_, *x*_2_) ∈ *ℕ* × ℝ^2^, where

(37) $$\begin{array}{c} a_{n}=\{\begin{array}{cc} e^{n(1+2^{n})} & \text{if}\;\;n=2k\\ e^{-(n+1)(1+2^{n+1})} & \text{if}\;\;n=2k+1. \end{array} \end{array}$$

Then for (*P*_*n*_) in *ℬ*(ℝ^2^) defined by

(38) $$\begin{array}{c} P_{n}\left(x_{1},x_{2}\right)=\left(x_{1},0\right) \end{array}$$

for all (*n*, *x*_1_, *x*_2_) ∈ *ℕ* × ℝ^2^, we have that for *α* = 1 and *D*(*n*) = *e* ^*n*(1+2^*n*^)^ the system ((*𝔄*)) is nonuniform exponentially dichotomic. Obviously, system ((*𝔄*)) is neither exponentially dichotomic nor uniformly exponentially dichotomic.

**Remark 22**

The system ((*𝔄*)) is nonuniform exponentially dichotomic with respect to the projections sequence (*P*_*n*_) invariant for ((*𝔄*)) if and only if there exist a constant *r* ∈ (0,1) and a function *D* : *ℕ* → [1, *∞*) such that

(39) $$\begin{array}{c} ||𝒜\left(m,n\right)x||\leq D\left(n\right)r^{m-n}||x||,\\ ||y||\leq D\left(m\right)r^{m-n}||𝒜\left(m,n\right)y||, \end{array}$$

for all (*m*, *n*) ∈ Δ and all (*x*, *y*) ∈ *Im*⁡*P*_*n*_ × Ker⁡*P*_*n*_.

A characterization of nonuniform exponential dichotomy of reversible systems is given by the following.

**Proposition 23**

The reversible system ((*𝔄*)) is nonuniform exponentially dichotomic if and only if there exist a projections sequence (*P*_*n*_) invariant for ((*𝔄*)), a function *D* : *ℕ* → [1, *∞*), and a constant *α* > 0 such that

(40) $$\begin{array}{c} ||𝒜\left(m,n\right)x||\leq D\left(n\right)e^{-\alpha (m-n)}||x||, \end{array}$$

(41) $$\begin{array}{c} ||{𝒜}^{-1}\left(m,n\right)y||\leq D\left(m\right)e^{-\alpha (m-n)}||y|| \end{array}$$

for all (*m*, *n*) ∈ Δ and all (*x*, *y*) ∈ *Im*⁡*P*_*n*_ × Ker⁡*P*_*m*_.

**Proof**

It is sufficient to prove the equivalence between (22) and (41).

*Necessity.* If (22) holds then for all (*m*, *n*, *y*) ∈ Δ × Ker⁡*P*_*m*_ we have

(42) ||𝒜(m,n)−1y|| =||𝒜(m,n)−1Qmy||=||Qn𝒜(m,n)−1Qmy|| ≤D(m)e−α(m−n)||𝒜(m,n)Qn𝒜(m,n)−1Qmy|| =D(m)e−α(m−n)||Qmy||=D(m)e−α(m−n)||y||.

*Sufficiency.* From (41) it results that for all (*m*, *n*, *y*) ∈ Δ × Ker⁡*P*_*m*_ we have

(43) ||y||=||Qny||=||𝒜(m,n)−1Qm𝒜(m,n)Qny||≤D(m)e−α(m−n)||Qm𝒜(m,n)Qny||=D(m)e−α(m−n)||𝒜(m,n)Qny||.

A characterization of nonuniform exponential dichotomy property with respect to strongly invariant projection sequences is given by the following.

**Theorem 24**

Let (*P*_*n*_) be a projections sequence which is strongly invariant for the system ((*𝔄*)). Then ((*𝔄*)) is nonuniform exponentially dichotomic with respect to (*P*_*n*_) if and only if there exist a function *D* : *ℕ* → [1, *∞*) and a constant *α* > 0 such that

(44) $$\begin{array}{c} ||𝒜\left(m,n\right)x||\leq D\left(n\right)e^{-\alpha (m-n)}||x||, \end{array}$$

(45) $$\begin{array}{c} ||ℬ\left(m,n\right)y||\leq D\left(m\right)e^{-\alpha (m-n)}||y|| \end{array}$$

for all (*m*, *n*) ∈ Δ and all (*x*, *y*) ∈ *Im*⁡*P*_*n*_ × Ker⁡*P*_*m*_.

**Proof**

It is sufficient to prove the equivalence between (22) and (45).

*Necessity.* We observe that from (22) and the properties (b_1_) and (b_3_) from Proposition 13, we obtain

(46) ||𝒜(m,n)y||  =||ℬ(m,n)Qmy||=(b3)||Qnℬ(m,n)Qmy||  ≤D(m)e−α(m−n)||𝒜(m,n)Qnℬ(m,n)Qmy||  =D(m)e−α(m−n)||Qm𝒜(m,n)ℬ(m,n)Qmy||  =(b1)D(m)e−α(m−n)||Qmy||=D(m)e−α(m−n)||y||,

for all (*m*, *n*, *y*) ∈ Δ × Ker⁡*P*_*m*_.

*Sufficiency.* By (45), using the property (b_2_) from Proposition 13 we obtain

(47) ||y||=||Qny||=(b2)||ℬ(m,n)𝒜(m,n)Qny||=||ℬ(m,n)Qm𝒜(m,n)Qny||≤D(m)e−α(m−n)||𝒜(m,n)Qny||=D(m)e−α(m−n)||𝒜(m,n)y||

for all (*m*, *n*, *y*) ∈ Δ × Ker⁡*P*_*n*_.

## 4. Lyapunov Functions and Nonuniform Exponential Dichotomies

Let ((*𝔄*)) be a linear discrete-time system on a Banach space *X* and (*P*_*n*_) a projections sequence which is invariant for ((*𝔄*)).

**Definition 25**

We say that *L* : Δ × *X* → ℝ_+_ is a Lyapunov function for the system ((*𝔄*)) with respect to projections sequence (*P*_*n*_) if there exists a constant *a* ∈ (1, +*∞*) such that the following properties hold:

(48) $$\begin{array}{c} L\left(m,n,x\right)-aL\left(m+1,n,x\right)\geq ||𝒜\left(m,n\right)x|| \end{array}$$

for all (*m*, *n*, *x*) ∈ Δ × *Im*⁡*P*_*n*_

(49) $$\begin{array}{c} L\left(m+1,n,y\right)-aL\left(m,n,y\right)\geq ||𝒜\left(m+1,n\right)y|| \end{array}$$

for all (*m*, *n*, *y*) ∈ Δ × Ker⁡*P*_*n*_,

(50) $$\begin{array}{c} L\left(n,n,y\right)\geq ||y|| \end{array}$$

for all (*n*, *y*) ∈ *ℕ* × Ker⁡*P*_*n*_.

**Example 26**

Let ((*𝔄*)) be the linear discrete-time system and (*P*_*n*_) the projections sequence considered in Example 11. Let

(51) $$\begin{array}{c} L\left(m,n,x\right)=2^{n-m}||P_{n}x||+2^{-m}e^{m-n}\overset{m}{\underset{k=n}{\sum }}2^{k}||Q_{k}x||, \end{array}$$

for all (*m*, *n*, *x*) ∈ Δ × *X*. By a simple computation for *a* = 2*e*/5 we can see that

(52) L(m,n,x)−2e5L(m+1,n,x)=(2−2e5)||𝒜(m,n)x||≥||𝒜(m,n)x||

for all (*m*, *n*, *x*) ∈ Δ × *Im*⁡*P*_*n*_ and

(53) L(m+1,n,y)−2e5L(m,n,y)≥em−n+1||Qm+1y||=||𝒜(m+1,n)y||

for all (*m*, *n*, *y*) ∈ Δ × Ker⁡*P*_*n*_.

Moreover

(54) $$\begin{array}{c} L\left(n,n,y\right)=||Q_{n}y||=||y|| \end{array}$$

for all (*n*, *y*) ∈ *ℕ* × Ker⁡*P*_*n*_.

The main result of this paper is as follows.

**Theorem 27**

The linear discrete-time system ((*𝔄*)) is nonuniform exponentially dichotomic with respect to the projections sequence (*P*_*n*_) invariant for ((*𝔄*)) if and only if there exists a nondecreasing sequence *β* : *ℕ* → [1, *∞*) such that

(55) $$\begin{array}{c} L\left(m,n,x\right)+L\left(m,n,y\right)\leq \beta \left(n\right)||x||+\beta \left(m\right)||𝒜\left(m,n\right)y|| \end{array}$$

for all (*m*, *n*) ∈ Δ and all (*x*, *y*) ∈ *Im*⁡*P*_*n*_ × Ker⁡*P*_*n*_.

**Proof**

*Necessity.* Suppose that ((*𝔄*)) is nonuniform exponentially dichotomic with respect to the projections sequence (*P*_*n*_). We define *L* : Δ × *X* → ℝ_+_ by

(56) L(m,n,x)=∑k=m∞dk−m||𝒜(k,n)Pnx|| +∑k=nmdm−k||𝒜(k,n)Qnx||,

where *d* ∈ (1, 1/*r*) and *r* is given by Remark 22. First, we observe that

(57) L(m,n,x)=∑k=m∞dk−m||𝒜(k,n)Pnx|| +∑k=nmdm−k||𝒜(k,n)Qnx||≤∑k=m∞dk−mD(n)rk−n||Pnx|| +∑k=nmdm−krm−kD(m)||𝒜(m,n)Qnx||≤D(n)1−dr||Pnx||+D(m)1−dr||𝒜(m,n)Qnx||=β(n)||Pnx||+β(m)||𝒜(m,n)Qnx||,

where *β*(*n*) = *D*(*n*)/(1 − *dr*), and thus (55) is verified.

On the other hand, for *x* ∈ *Im*⁡*P*_*n*_ we have that

(58) L(m,n,x)=∑k=m∞dk−m||𝒜(k,n)x||=d0||𝒜(m,n)x||+d1||𝒜(m+1,n)x||+⋯=amn+dam+1n+d2am+2n+⋯,

where *a*_*m*_^*n*^ = ||*𝒜*(*m*, *n*)*x*||. Moreover

(59) L(m+1,n,x) =∑k=m+1∞dk−m−1||𝒜(k,n)x|| =d0||𝒜(m+1,n)x||+d1||𝒜(m+2,n)x||+⋯ =am+1n+dam+2n+d2am+3n+⋯.

From (58) and (59) we have that

(60) $$\begin{array}{c} L\left(m,n,x\right)=a_{m}^{n}+dL\left(m+1,n,x\right). \end{array}$$

Hence

(61) $$\begin{array}{c} L\left(m,n,x\right)-aL\left(m+1,n,x\right)\geq ||𝒜\left(m,n\right)x||, \end{array}$$

for all (*m*, *n*, *x*) ∈ Δ × *Im*⁡*P*_*n*_.

In the same manner we can see that for *y* ∈ Ker⁡*P*_*n*_ we have that

(62) L(m+1,n,y)=∑k=nm+1dm−k||𝒜(k,n)y||=dL(m,n,y)+||𝒜(m+1,n)y||.

Hence

(63) $$\begin{array}{c} L\left(m+1,n,y\right)-aL\left(m,n,y\right)\geq ||𝒜\left(m+1,n\right)y||. \end{array}$$

*Sufficiency.* According to (48) for every (*m*, *n*, *x*) ∈ Δ × *Im*⁡*P*_*n*_ we have that

(64) $$\begin{array}{c} L\left(n,n,x\right)-aL\left(n+1,n,x\right)\geq ||𝒜\left(n,n\right)x||\\ L\left(n+1,n,x\right)-aL\left(n+2,n,x\right)\geq ||𝒜\left(n+1,n\right)x||\\ \vdots \end{array}$$

which implies

(65) L(n,n,x)≥∑j=n∞aj−n||𝒜(j,n)x||=∑k=0∞ak||𝒜(n+k,n)x||.

From (65) we have that

(66) $$\begin{array}{c} a^{m-n}||𝒜\left(m,n\right)x||\leq L\left(n,n,x\right)\leq \beta \left(n\right)||x||. \end{array}$$

Hence

(67) $$\begin{array}{c} ||𝒜\left(m,n\right)x||\leq {\left(\frac{1}{a}\right)}^{m-n}\beta \left(n\right)||x|| \end{array}$$

for all (*m*, *n*, *x*) ∈ Δ × *Im*⁡*P*_*n*_.

In a completely analog way, from (49) and (50) for *y* ∈ Ker⁡*P*_*n*_ we have that

(68) $$\begin{array}{c} L\left(m,n,y\right)-aL\left(m-1,n,y\right)\geq ||𝒜\left(m,n\right)y||\\ L\left(m-1,n,y\right)-aL\left(m-2,n,y\right)\geq ||𝒜\left(m-1,n\right)y||\\ \vdots \\ L\left(n+1,n,y\right)-aL\left(n,n,y\right)\geq ||𝒜\left(n+1,n\right)y||\\ \;\;\;\;L\left(n,n,y\right)\geq ||y|| \end{array}$$

which implies

(69) L(m,n,y)≥∑j=nmam−j||𝒜(j,n)y||≥am−n||𝒜(n,n)y||=am−n||y||.

By (69) we have

(70) $$\begin{array}{c} a^{m-n}||y||\leq L\left(m,n,y\right)\leq \beta \left(m\right)||𝒜\left(m,n\right)y||. \end{array}$$

Hence

(71) $$\begin{array}{c} ||y||\leq \frac{\beta \left(m\right)}{a^{m-n}}||𝒜\left(m,n\right)y|| \end{array}$$

for all (*m*, *n*, *y*) ∈ Δ × Ker⁡*P*_*n*_. From (67) and (71) we obtain that system ((*𝔄*)) is nonuniform exponentially dichotomic. Thus, the proof is completed.

**Corollary 28**

The linear discrete-time system ((*𝔄*)) is exponentially dichotomic with respect to the projections sequence (*P*_*n*_) if and only if there exist some constants *K*, *p* ≥ 1 and a Lyapunov function *L* with respect to (*P*_*n*_) such that

(72) $$\begin{array}{c} L\left(m,n,x\right)+L\left(m,n,y\right)\leq K\left(p^{n}||x||+p^{m}||𝒜\left(m,n\right)y||\right) \end{array}$$

for all (*m*, *n*) ∈ Δ and all (*x*, *y*) ∈ *Im*⁡*P*_*n*_ × Ker⁡*P*_*n*_.

**Corollary 29**

The linear discrete-time system ((*𝔄*)) is uniformly exponentially dichotomic with respect to the projections sequence (*P*_*n*_) if and only if there exist a Lyapunov function with respect to (*P*_*n*_) and a constant *K* ≥ 1 such that

(73) $$\begin{array}{c} L\left(m,n,x\right)+L\left(m,n,y\right)\leq K\left(||x||+||𝒜\left(m,n\right)y||\right) \end{array}$$

for all (*m*, *n*) ∈ Δ and all (*x*, *y*) ∈ *Im*⁡*P*_*n*_ × Ker⁡*P*_*n*_.
